# The Influence of Oxidation and Nitrogenation on the Physicochemical Properties and Sorption Capacity of Activated Biocarbons Prepared from the Elderberry Inflorescence

**DOI:** 10.3390/molecules28145508

**Published:** 2023-07-19

**Authors:** Wiktoria Dąbrowska, Mateusz Gargol, Małgorzata Gil-Kowalczyk, Piotr Nowicki

**Affiliations:** 1 Department of Applied Chemistry, Faculty of Chemistry, Adam Mickiewicz University in Poznań, Uniwersytetu Poznańskiego 8, 61-614 Poznań, Poland; 2 Laboratory of Optical Fibers Technology, Faculty of Chemistry, Institute of Chemical Sciences, Maria Curie-Sklodowska University, M. Curie-Sklodowska Sq. 5, 20-031 Lublin, Poland

**Keywords:** elderberry herb, activated biocarbons, physical activation, chemical activation, adsorption, methylene blue, rhodamine B

## Abstract

The main objective of the study was to prepare a series of new activated biocarbons by means of physical and chemical activation of elderberry inflorescence. The influence of carbon matrix nitrogenation/oxidation on the physicochemical properties and sorption abilities of the carbonaceous materials was investigated. The impact of initial dye concentration, pH and temperature of the system on methylene blue and rhodamine B removal efficiency was checked. It was shown that activation of elderberry inflorescences with CO_2_ or H_3_PO_4_, and their further modification by introducing nitrogen or oxygen functional groups, allowed us obtain a wide range of materials that differ significantly in terms of the chemical nature of the surface, degree of specific surface development and the type of porous structure generated. The samples prepared by chemical activation proved to be very effective in terms of cationic dyes adsorption. The maximum sorption capacity toward methylene blue and rhodamine B reached the level of 277.8 and 98.1 mg/g, respectively. A better fit to the experimental data was achieved with a Langmuir isotherm than a Freundlich one. It was also shown that the efficiency of methylene blue and rhodamine B adsorption from aqueous solutions decreased with increasing temperature of the system.

## 1. Introduction

Activated carbons/biocarbons are carbonaceous materials with the unique physical and chemical properties, thanks to which they are widely used in various branches of modern industry [1]. They also play a very important role in water and wastewater treatment as well as in the broadly understood environmental protection [2]. Activated carbons are very effective, universal and, at the same time, relatively cheap adsorbents, which is why they are commonly used for the removal of various organic and inorganic pollutants, both from the gas and liquid phase. In the literature on the subject, there are numerous reports on their application for the adsorption of such pollutants as: toxic inorganic gases (for example, NH_3_, NO_2_, H_2_S, SO_2_, CO_2_) [3,4], volatile organic compounds (VOCs) [5], heavy metal ions [6,7], phenol and its derivatives [8], organic dyes [9,10], pharmaceuticals [11], surfactants [12], polymers [13], etc. Furthermore, activated carbons are increasingly used as catalysts or catalyst support [14], as electrode materials for electrochemical capacitors and lithium-ion batteries [15], as well as materials for gas storage [16].

There are many ways of obtaining activated carbons, which differ quite significantly in the procedure, but each of them is aimed at a significant development of the specific surface area and the formation of a polydisperse porous structure, because these parameters mainly determine the unique properties of carbon materials. The two most popular methods are two-step physical activation using gaseous activators (CO_2_, steam or a mixture of both) and one-step chemical activation of the precursor using reagents such as ZnCl_2_, H_3_PO_4_, KOH, K_2_CO_3_, etc. [17]. Fossil coals, peat, wood and coconut shells are most often used as precursors for the production of activated carbons [18]. However, due to economic and ecological reasons, waste biomass is increasingly used for this purpose, including fruit stones, nut shells, sawdust, corn cobs and various types of agricultural waste [19,20,21,22,23,24]. Such a solution becomes very important in the era of today’s ecological and energy crisis. The production of activated biocarbons is an excellent alternative to the storage or disposal of waste by incineration. Furthermore, the products obtained in this way can be used to clean sewage, air and soil from various types of organic and inorganic pollutants, thus limiting the degradation of the natural environment.

Recently, much attention was devoted to the carbonaceous materials modified to contain different heteroatomic functional groups or metal ions in their structure. Modification of the precursors or activation products via nitrogenation, oxidation, impregnation or plasma and heat treatment permits obtaining materials of specific acidic–basic nature of the surface, hydrophobic–hydrophilic character as well as improved adsorption or catalytic properties [25,26,27,28,29,30,31,32]. Particularly promising are materials enriched in nitrogen and oxygen functional species because of wide possibilities of their application, both in various branches of industry as well as in environment protection [33,34,35,36,37], electrochemistry [15,38,39] and even modern medicine [32].

Taking the above into account, the main objective of this work was to prepare a series of new activated biocarbons via direct physical and chemical activation of herbal industry waste—elderberry inflorescence (residues from the production of dietary supplements, herbal teas, etc.) as well as to assess their usefulness for the removal of organic dyes from the aqueous solutions. These highly toxic organic compounds (in many cases, suspected of carcinogenic or mutagenic activity) are widely used in the textile, paper, plastics, cosmetics, leather and food industries. The dyes contained in wastewater pose a serious threat to the quality of water resources, and taking into account their bioaccumulation, these pollutants can reach people through the food chain. Therefore, monitoring their quantity as well as effective removal from wastewater is very important.

In addition, the effect of two variants of post-activation modification (oxidation with HNO_3_ or nitrogen introduction via reaction with urea) on the chemical nature of the surface, textural parameters, thermal stability as well as adsorption capacity of the obtained activated biocarbons was investigated.

## 2. Results and Discussion

### 2.1. Elemental Composition of the Starting Elderberry Inflorescence as Well as Activated Biocarbons Obtained via Its Physical/Chemical Activation and Further Thermochemical Modification

According to the data presented in Table 1, the elderberry inflorescence used for the study was characterized by a fairly high share of elemental carbon (49.1 wt.%). Unfortunately, almost 1/10 of its structure was mineral admixtures (so-called ash), the presence of which was not desirable (especially from an adsorption point of view). Noteworthy is also the high content of nitrogen in the starting material (nearly 3 wt.%).

Further analysis of the obtained data showed that both activation procedures led to intensive changes in the elemental composition in relation to the precursor. As a result of thermal treatment in the presence of carbon dioxide (sample P) or orthophosphoric acid (sample C), the content of elemental carbon increased significantly, while the share of other elements (in particular oxygen and hydrogen) was clearly reduced. However, the intensity of these changes depends to a large extent on the variant of the activation procedure, which is related to a different mechanism of reaction between waste biomass and CO_2_ or H_3_PO_4_. As a result of thermochemical conversion of elderberry inflorescence, the content of mineral admixtures in the carbonaceous structure also changed. In case of the sample obtained via direct physical activation of the precursor, it increased almost threefold. In turn, for analogous material activated with orthophosphoric acid, a slight decrease was observed. Such a big difference between the activation products resulted from a diverse chemical nature of both activating agents as well as procedure of thermal treatment applied. Physical activation was carried out at a higher temperature (700 °C), which resulted in the oxidation and/or distillation of greater amount of volatile components present in the structure of the starting material, and, thus, the share of the mineral substance significantly increased. During the chemical activation, the processes of dehydration, degradation and condensation of the starting material took place, accompanied by the release of carbon oxides. In addition, the reaction of H_3_PO_4_ with the cellulose present in the precursor may result in the formation of phosphate esters. However, due to the lower processing temperature, the loss of organic components was less intense than for activation with CO_2_; therefore, the share of mineral substance in the structure of the activation product was lower. Furthermore, during the procedure of direct activation, there was no additional stage of the final product purification (which takes place in the case of chemical activation), when the ballast substances had a chance to be partially removed during washing with hot distilled water. Furthermore, earlier impregnation of the precursor with H_3_PO_4_ before the thermal treatment stage also had a positive effect on the process of cleaning the structure from non-carbon impurities. Similar dependencies were observed in the case of activation of other precursors, such as post-fermentation residue [40], mugwort stems and leaves [41] or low-quality brown coals [42].

The data presented in Table 1 show that both variants of modifications carried out after the activation process led to a significant reduction in the ash content. A much more spectacular effect was achieved in case of the oxidation procedure with nitric acid. This effect was particularly evident in case of the POX sample, where the ash content was reduced almost tenfold. During the oxidation procedure, the activated biocarbon sample stays in boiling nitric acid for 3 h; as a result, a significant part of the inorganic matter can undergo digestion. This allowed for unblocking of activated biocarbon porous structure and removal of mineral admixtures. In case of modification by nitrogenation, the sample was annealed in the presence of urea, and then rinsed with hot distilled water, which allowed the removal of a small amount of inorganic impurities contained in the carbonaceous structure.

Both of the applied post-activation modifications led also to significant changes in the elemental composition of activated biocarbons. After modification by means of oxidation, the content of elemental carbon clearly decreased, which was accompanied by a significant increase in the share of oxygen in the structure. These changes were definitely greater in case of the COX sample. In turn, as a result of the nitrogenation procedure, C^daf^ contribution in the carbon structure increased. In case of the CN sample, this increase was admittedly small (only by 0.3 wt.%), while for the PN sample, it was as much as 7.6% by weight. In contrast to oxidation process, annealing of activated biocarbons in the presence of urea (especially in case of the product of physical activation) led to a decrease in the oxygen content in the structure. Interestingly, both variants of modification contribute to the increase in the share of nitrogen in the carbon matrix; however, the samples subjected to reaction with urea were characterized by a higher content of this heteroatom.

### 2.2. Acidic-Basic Properties of the Precursor and the Activated Biocarbons Prepared

An analysis of the data summarized in Table 2 showed that the precursor selected for the study contained significant amounts of acidic and basic functional groups, which is quite typical for plant biomass. However, activated biocarbons obtained as a result of direct physical and chemical activation of elderberry inflorescence showed a completely different acidic–basic character of the surface, which may be important from the perspective of their future practical application. Only basic functional groups were present on the surface of the sample activated with CO_2_, and the pH value of its aqueous extract was almost 11. This was a consequence of the very high ash content in the structure of this sample (over 26 wt.%), which is usually alkaline in nature. On the contrary, in case of the sample obtained via chemical activation of elderberry inflorescence with H_3_PO_4_, a clear predominance of acidic groups (1.18 mmol/g) over basic ones (0.60 mmol/g) was observed, and its pH value was only 2.62, which was the lowest value among all carbon materials under study. These results are further evidence that confirm the different mechanism of interaction between the activating agents applied and the plant material selected as the activated biocarbon’s precursor.

Nitrogenation of the P sample slightly lowered the pH of the aqueous extract, which was probably a consequence of the introduction of a small amount of acidic groups (0.15 mmol/g) and a simultaneous reduction in the proportion of groups of basic nature. However, it should be emphasized that despite a significant decrease, the content of basic functional groups was still very high, i.e., 4.17 mmol/g. In turn, the oxidation of biocarbon obtained as a result of direct physical activation caused a threefold decrease in pH. Such a low pH value in the case of the POX sample (3.65) was a consequence of the introduction of a significant amount of acidic functional groups into the carbon matrix (an increase of 2.72 mmol/g) and a drastic decrease in the share of basic functional species from 5.28 mmol/g to as little as 0.90 mmol/g. A decrease in the content of the latter was most likely the result of the removal of a significant part of inorganic impurities present in the structure of the P sample as a result of reaction with hot HNO_3_ (the effect of partial demineralization of the sample).

“C”-series biocarbons generally have quite low pH values, with the highest score being slightly above 6.1 for sample CN. Oxidation of the chemical activation product (similarly as for the P sample) increased the contribution of acidic groups, but the difference between the COX and C samples was only 0.09 mmol/g. Nevertheless, the pH value of the aqueous extract of the oxidized sample was higher (4.67) than for the initial sample (2.62), which resulted from the fact that after modification, the content of basic groups also increased quite significantly. In turn, as a result of nitrogenation of the C sample, the content of basic functional groups increased more than twice, which was accompanied by a significant (over 50%) reduction in the share of acidic groups.

### 2.3. Textural Parameters of the Activated Biocarbons Obtained from Elderberry Inflorescence

**Figure 1:**
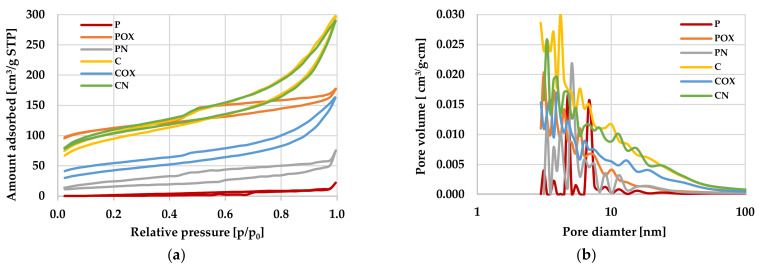
Low-temperature nitrogen adsorption/desorption isotherms (**a**) as well as pore size distributions (**b**) for the activated biocarbons prepared from the elderberry inflorescence.

The analysis of the data presented in Table 3 and Figure 1 showed that the carbon adsorbents obtained as a result of physical and chemical activation of the elderberry inflorescence were characterized by very diverse textural parameters. At first glance, it can be seen that the product of direct physical activation (P sample) was much less favourable in terms of texture. Its specific surface area was only 2 m^2^/g, while the total pore volume was less than 0.035 cm^3^/g. Moreover, this material did not contain micropores in its structure, and the average pore diameter was as much as 67.28 nm, so it can be practically considered a non-porous material. Such poor textural parameters were most probably a consequence of the very high ash content in sample P (Table 1), which blocked a significant part of the pores present in its structure. The product of chemical activation showed much better textural parameters, because its specific surface area slightly exceeded 316 m^2^/g and the total pore volume was 0.46 cm^3^/g (of which 15% are micropores). However, this was not a fully satisfactory result for this activation variant. For example, activated carbons obtained via chemical activation of such precursors as: wheat bran [43], reed straw [44], lotus seed pods [45], acacia wood [46] or miscanthus and switchgrass [47] achieved specific surface area and total pore volume at the level of 339–1796 m^2^/g and 0.13–1.52 cm^3^/g, respectively. Therefore, the procedure for the production of activated biocarbons from elderberry inflorescences required further optimisation.

Analysis of the data summarized in Table 3 clearly indicate that both of the post-activation modifications significantly changed the textural parameters of the activated biocarbons obtained from elderberry inflorescence. However, it should be noted here that the effect of oxidation and nitrogenation of the product of physical and chemical activation was quite different. Oxidation of the sample activated with CO_2_ resulted in a spectacular increase in the specific surface area (by 340 m^2^/g), as well as the eight-fold increase in the total pore volume and a more than twenty-fold decrease in the average pore size. Such favourable changes were most probably a consequence of the dissolution of a significant part of mineral admixtures present in the structure of sample P as a result of reaction with hot HNO_3_ and their subsequent washing with hot distilled water. Modification of the P sample via nitrogenation also brought a beneficial effect in the context of textural parameters, but its scale was definitely smaller (S_BET_ = 56 m^2^/g, V_T_ = 0.117 cm^3^/g).

On the contrary, as a result of the C sample oxidation, a more than two-fold decrease in the specific surface area and pore volume was observed, which was accompanied by an increase in the average pore diameter and a significant decrease in the micropores contribution in the total pore volume. This was a consequence of the incorporation of significant amounts of oxygen functional groups into the carbon matrix, which made it difficult for adsorbate molecules (liquid nitrogen) to access the smaller pores during low-temperature nitrogen adsorption/desorption measurements. The reason for the deterioration of the textural parameters could also be too drastic conditions of the oxidation procedure, as a result of which some of the micropores were enlarged to mesopores, as indicated by the increase in the average pore size by more than 1 nm. The opposite tendency of changes in textual parameters was observed in the case of the CN sample, for which the specific surface area increased by 16.8 m^2^/g, the average pore width decreased to 5.389 nm and the share of micropores in the structure increased by about 4%.

The data discussed above (Table 3) were additionally illustrated in Figure 1 presenting the isotherms of N_2_ adsorption/desorption on the activated biocarbons as well as the pore size distribution curves for the materials under investigation. The shape of isotherms (Figure 1a) confirmed significant differences between the porous structure of carbons activated with CO_2_ and H_3_PO_4_. The isotherms obtained for the P and PN biocarbons had a very irregular shape (which is quite typical for materials with a poorly developed porous structure) and it was difficult to determine their type according to the IUPAC classification. In case of the remaining activated biocarbon samples, the isotherms were smooth and their shape was similar to the IV type, characteristic of solids containing significant amounts of mesopores in the structure. Moreover, in the course of these isotherms, the wide hysteresis loops were observed (especially for the POX sample), which also confirmed the predominance of mesopores in the total pore volume. The final confirmation of the significant textural diversity of individual carbon materials were the pore size distribution curves presented in Figure 1b. According to these data, the “C”-series samples showed significantly higher values of pore volume, both in the range of small mesopores (diameter of 2–5 nm) as well as of large mesopores (size in the range of 10–50 nm).

### 2.4. Thermal Propeties of the Activated Biocarbons Prepared from Elderberry Inflorescence

**Figure 2:**
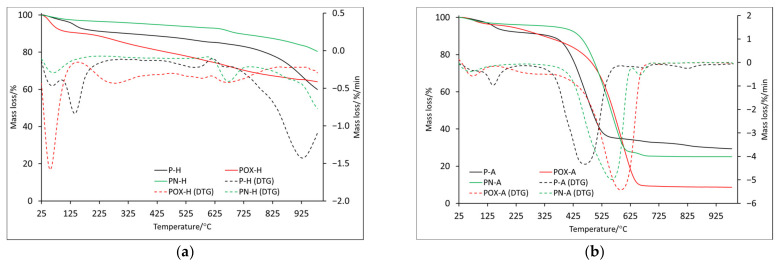
The TG and DTG curves for samples obtained by physical activation: in helium atmosphere (**a**) and air atmosphere (**b**).

**Figure 3:**
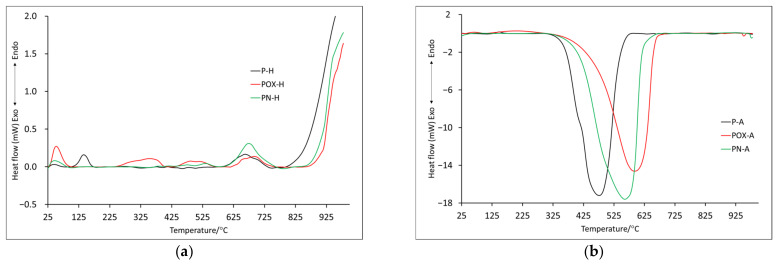
The DSC curves for samples obtained by physical activation: in helium atmosphere (**a**) and air atmosphere (**b**).

In order to fully characterize the thermal properties of the prepared materials, thermal analyses were performed in two atmospheres: helium and air (symbols H and A, respectively). The TG (thermogravimetry) and DTG (differential thermogravimetry) curves obtained for samples after physical activation are presented in Figure 2a (helium) and Figure 2b (air). In order to better understand the processes taking place, measurements and analysis of the results obtained from differential scanning calorimetry (DSC) were also performed (Figure 3). The most important data of these measurements are collected in Table 4 and Table 5.

By comparing the course of the TG and DTG curves (Figure 2a), several similarities can be observed. The first recorded mass loss (T1), the maximum of which for all samples was below 100 °C (Table 4), was most likely related to the presence of water physically adsorbed on the surface. The hygroscopic properties of the analyzed carbons should be taken into account, which, based on the mass loss value, would indicate that the POX-H sample showed the greatest properties in question. For comparison, the POX-H sample at 100 °C showed almost 9% mass loss, while the P-H and PN-H samples showed only 3% and 2.2%, respectively. The next two mass losses were recorded only for the P-H and POX-H samples; however, the T2 temperature for the oxidized sample (POX-H) was shifted towards a higher value (140.7 °C and 276.0 °C, respectively), while the T3 values for these samples were very similar. Around the temperature of 670 °C, a change in mass was recorded for all samples (T4). Based on the RM (residual mass, Table 4) value, it can be unequivocally stated that the PN-H sample showed the best thermal stability, as the mass loss recorded at 1000 °C was only 19.8%. The POX-H sample, at the same temperature, showed the mass loss of 36.3%, while the P-H sample showed the mass loss of 40.3%. At the same time, it is worth noting that the mass loss for the POX-H sample was rather slow and uniform, starting from the temperature of about 325 °C. In the case of the P-H sample, a rather rapid increase in mass loss was recorded around 700 °C, with two maximums in temperature 786.1 °C—T5 and 931.3 °C—T6 (which probably indicates a more complex decomposition mechanism).

It is also worth comparing ITD (initial thermal decomposition), at which the final decomposition of the carbonaceous material begins. It was assumed that ITD was the initial temperature of the last recorded mass change. On its basis, it can be concluded that the oxidized sample, the ITD of which was recorded at the highest temperature (850.0 °C) and for which, as already mentioned, the mass loss was rather slow and uniform, showed the highest thermal stability. The lowest value of ITD was recorded for the P-H sample, while the value was quite high for the nitrogenated sample (746.5 °C). Data obtained from DSC (Table 5) indicated that all recorded mass changes were related to endothermic processes. Some mass losses, which in the DTG curve showed one maximum, in the DSC curves showed two maximums (D2-D4), which proved the complexity of the decomposition process. Based on DSC data, it was also possible to indicate the ITD, which, in this case, was the initial temperature at which the last energy change was recorded. For the PN-H and POX-H samples, the IDT values were very similar at 865.2 °C and 861.9 °C, respectively. The IDT for the P-H sample was slightly lower (772.9 °C), indicating that it had the least thermal stability.

The onset of mass changes for samples analyzed in air was quite similar to this recorded in the helium atmosphere. The maximum of the first mass loss (T1) for all samples was recorded below 100 °C (Table 4) and their values were also similar (P-A: 1.6%, POX-A: 2.8%, PN-P: 2.3%), only for the oxidized sample was it lower. All the samples were annealed before being analyzed; however, a small amount of water remained, although it is more likely that because it took about 2 h from the time the sample was taken out of the vial to the start of the analysis, during which the apparatus stabilized and water was reabsorbed. The second mass change (T2) was observed only for the P-A and POX-A samples. As in the case of the helium atmosphere, the maximum mass loss recorded for the oxidized sample was shifted towards the higher temperature (271.6 °C). It is worth noting that the T2 values for the helium and air atmospheres were very similar (Table 4). Up to this point, the observed mass changes were related to endothermic processes, the mass loss T3 for all samples was recorded as an exothermic process (Table 5). This, of course, was associated with the carbon combustion in the air atmosphere. Additionally, that is why the beginning of this process was acknowledged as ITD, with values: 257.9 °C, 364.8 °C and 286.7 °C for samples P-A, POX-A and PN-A, respectively. On their basis, it can be concluded that the oxidized sample showed better thermal stability. It is also worth noting that the DTG curve, in the section corresponding to the T3 mass loss for the P-A and PN-A samples, was slightly wider than in the case of the oxidized sample. This resulted in the possibility of determining two maxima for this stage of decomposition (407.1, 465.3 for sample P-A and 485.0, 559.7 for sample PN-A). 

It is worth noting that on the DSC curve, the recorded mass changes also corresponded to two maxima of exothermic effects (Table 5). In addition, the T3 values moved towards higher temperatures starting from the P-A sample and ending with the POX-A sample, which further confirmed the fact that the oxidized sample showed better thermal properties (Table 4). For the P-A and PN-A samples, further mass changes (T4 and T5) were recorded, which were most likely related to the combustion of inorganic residues, the content of which was higher than in the case of the oxidized sample, as indicated by the residual mass (Table 4). As for the ITD recorded on the DSC curve basis (beginning of the proper thermal degradation D3), it reached similar values for all analyzed samples.

**Figure 4:**
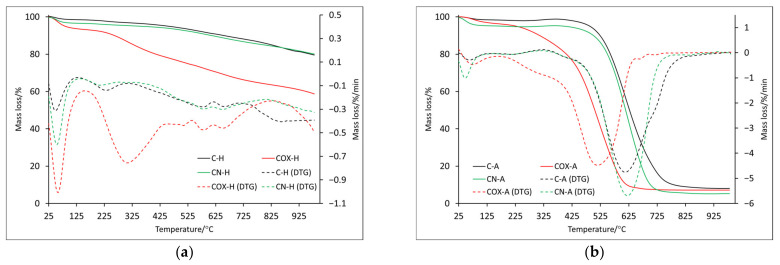
The TG and DTG curves for samples obtained by chemical activation: in helium atmosphere (**a**) and air atmosphere (**b**).

**Figure 5:**
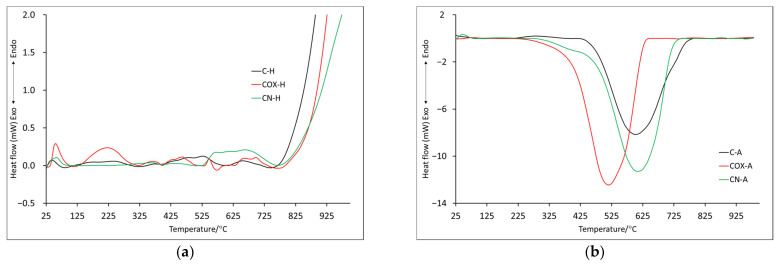
The DSC curves for samples obtained by chemical activation: in helium atmosphere (**a**) and air atmosphere (**b**).

Just as it was in case of the physically activated samples, an endothermic process occurred associated with the evaporation of surface-adsorbed water in case of the chemically activated materials (analyzed both in helium and in air atmosphere) below 100 °C (T1/D1) (Figure 4 and Figure 5). Looking at the mass loss values recorded at the 100 °C (C-H: 1.4%, COX-H: 5.7%, CN-H: 3.2%, C-A: 1.4%, COX-A: 2.3%, CN-A:5.6%), we can see that they were not much different from those recorded for the physically activated samples, a slight increase in values was noticeable for the CN-H and CN-A samples.

The maximum of the second mass loss (Table 6) is in the temperature range of about 210–310 °C, with the highest value for oxidized sample (COX-H: 307.9 °C). In this case, if any effect was registered on DSC curves, it was endothermic (Figure 5). Subsequent mass losses (T3–T5) for samples analyzed in the helium atmosphere are related to the thermal decomposition of inorganic groups introduced into the sample during its processing. The discussed mass changes were associated with endothermic processes (Figure 5a, Table 7), but it is worth noting that for the nitrogenated sample, several maxima were recorded, which proved a more complex process of thermal decomposition. ITD takes higher values than in the case of identical analysis only for physically activated samples. Only for POX-H and COX-H samples, these values were very similar (850.0 °C and 846.3 °C, respectively) and at the same time, they were the largest, which proved better thermal resistance. Based on the residual mass, we can see that the oxidized sample underwent thermal decomposition to the greatest extent (Table 6).

It was also noticeable that for the P-H sample, the residual mass value was 20% lower compared to the C-H sample. Therefore, it should be taken into account that due to physical activation, the sample surface changed significantly, but this relationship was not noticeable for nitrogenated or oxidized samples. For samples analyzed in air, the T3 mass loss reached two maxima (three for sample C-A, Table 6). In addition, a similar decomposition course of samples C-A and CN-A was noticeable, two maxima of which were recorded at almost identical temperatures (397.5 °C, 613.0 °C and 400.5 °C, 622.9 °C, respectively). For the oxidized sample, the two maxima were shifted towards lower temperatures, which proved that the thermal stability was lower. This was further confirmed by the ITD, which had the lowest value for COX-A sample (161.3 °C). In the case of the mineral residue, it was comparable in all cases, slightly lower for the nitrogenated sample (but the values were lower than case of the physically activated samples, which may indicate a greater impact of chemical activation on the samples surface). Of course, the energy transformations that occurred for the T3 and T4 mass loss were related to the combustion of the samples, which was associated with the energy release (Table 7). The ITD values determined from the DSC curves for samples analyzed in helium were quite similar (between 760 and 780 °C), while samples analyzed in air were related to the beginning of the proper decomposition marked as D3 in Table 7 (C-A: 427.2 °C, COX-A: 232.2 °C, CN-A: 285.7 °C, respectively).

### 2.5. Sorption Performance of the Activated Biocarbons Prepared from Elderberry Inflorescence in Relation to Methylene Blue (MB) and Rhodamine B (RhB)

**Figure 6:**
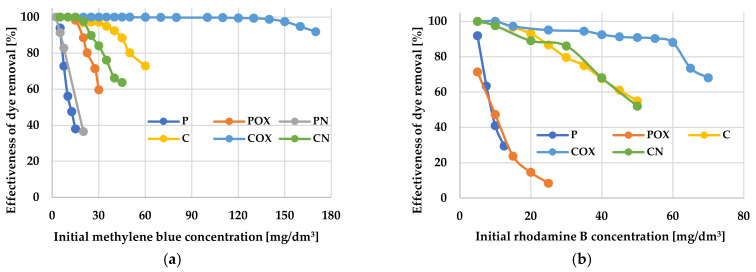
The effectiveness of methylene blue (**a**) and rhodamine B (**b**) removal from aqueous solution.

**Figure 7:**
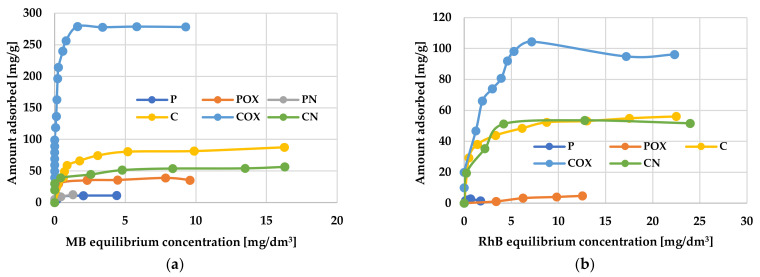
The equilibrium isotherms of methylene blue (**a**) and rhodamine B adsorption (**b**) on the activated biocarbons prepared from elderberry inflorescence.

The results of adsorption tests presented in Table 8 and Table 9 and in Figure 6 and Figure 7 indicate unequivocally that the products of physical and chemical activation of the elderberry inflorescence, as well as the carbonaceous materials obtained as a result of their thermochemical modification, showed very diverse efficiency of organic dyes removal. Each of the tested materials was able to adsorb a greater amount of methylene blue than of rhodamine B. This was most probably related to the size of the RhB molecules, which were much more spatially expanded than the MB ones. However, it should be emphasized that the adsorption capacities of the samples obtained as a result of chemical activation (“C”-series) were significantly higher.

The most effective adsorbent was the COX sample, which sorption capacity towards both organic pollutants was significantly higher than for the other activated biocarbons. This was particularly visible in case of methylene blue removal, where the difference between the COX sample (q_e_ = 278.99 mg/g) and the least effective adsorbent—P sample (q_e_ = 11.75 mg/g) was as much as 267.24 mg/g. The COX sample was also characterized by the highest efficiency of MB and RhB removal from the liquid phase. According to the data presented in Figure 6a, this sample showed almost 100% efficiency in methylene blue removal in a very wide range of its initial concentrations, i.e., ranging from 5 to 130 mg/dm^3^. Moreover, a further increase in the initial concentration of MB to the level of 170 mg/dm^3^ resulted in a 10% decrease in adsorption efficiency. In case of the rhodamine B adsorption, the result was much less impressive, because 100% efficiency of dye removal was observed for its initial concentrations ranging from 5 mg/dm^3^ to 20 mg/dm^3^. Unfortunately, in case of the other biocarbons (in particular, for CO_2_-activated samples), the adsorption efficiency was definitely lower. The least favourable, in this respect, were the P and PN samples, which did not reach 100% efficiency even at the lowest concentrations of both dyes (below 5–10 mg/dm^3^). This ruled out the possibility of their practical application as potential adsorbents of organic pollutants. 

Following the analysis of the data collected in Table 8 and Table 9, modification of activated biocarbons by means of nitric acid oxidation had a positive effect on their sorption capacity towards cationic organic dyes. This was most probably a consequence of the introduction of significant amounts of functional groups on the carbonaceous adsorbents surface, which can interact with molecules of methylene blue and rhodamine B (e.g., through electrostatic interactions, hydrogen bonding). Unfortunately, the introduction of nitrogen functional groups was not conducive to improving the sorption ability of activated biocarbons. The only exception was the PN sample, which was able to adsorb slightly more methylene blue than unmodified carbon.

A very important factor determining the effectiveness of removing organic pollutants from aqueous solutions was the initial concentration of the adsorbate, because it was the driving force of the diffusive and mass transfer processes involved in adsorption process [48]. According to the data presented in Figure 7, the amounts of adsorbed methylene blue and rhodamine B increased significantly with increasing initial concentration of these dyes in the aqueous solution. This suggests that at lower cationic dye concentrations, their adsorption on the activated biocarbons surface is accidental. When the initial MB and RhB concentration increased, the number of collisions between the dye molecules and the activated biocarbons surface also increased, thus improving the effectiveness of adsorption process. In turn, at higher dye concentrations, all the active centers located on the adsorbents surface were completely occupied by MB or RhB molecules, and the surface/porous structure of the carbonaceous materials was fully saturated. Moreover, the smooth shape of the isotherms may indicate the monolayer coverage of the activated biocarbons surface with the dye molecules.

**Figure 8:**
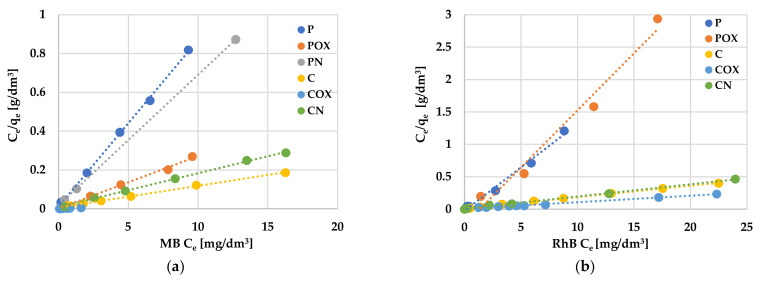
Linear fitting of methylene blue (**a**) and rhodamine B adsorption isotherms (**b**) on activated biocarbons to Langmuir model.

**Figure 9:**
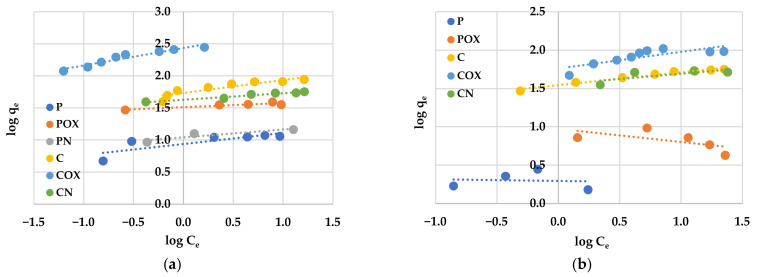
Linear fitting of methylene blue (**a**) and rhodamine B adsorption isotherms (**b**) on activated biocarbons to Freundlich model.

An analysis of experimental data using various adsorption models allowed us to explain how the carbonaceous adsorbent interacts with the adsorbate molecules. According to the data presented in Table 8 and Table 9 and in Figure 8 and Figure 9, the Langmuir model isotherm fit the experimental data more accurately than the Freundlich one. The value of the determination coefficient (R^2^) for this model was definitely closer to unity, both for methylene blue (0.9883–0.9995) and rhodamine B sorption (0.8901–0.9982). Moreover, the calculated values of q_m_ were very close to the sorption capacities determined experimentally (q_e_). Therefore, it can be assumed that MB and RhB adsorption proceeded with the formation of the adsorbate monolayer on the activated biocarbon surface (each dye molecule had equal activation energy and there were no interactions between adsorbed molecules). However, the high values of R^2^ for the Freundlich isotherm model observed in case of the COX (0.9235) and CN samples (0.9558, Table 8) as well as the for the C sample (0.9726, Table 9) suggested that the adsorption mechanism of these dyes was much more complicated and may also include multilayer adsorption, i.e., interactions between adsorbate molecules. In addition, the constant 1/n was significantly lower than 1.0, indicating that methylene blue and rhodamine B molecules were favourably adsorbed by the activated biocarbons prepared.

Adsorption tests were also carried out for a mixture of both dyes, using the following three measurement variants: (1) much higher initial concentration of methylene blue than rhodamine B, (2) equal concentrations of both dyes, (3) much higher concentration of RhB than MB. In order to better illustrate the effect of the second dye presence on the activated biocarbons sorption capacity towards both organic pollutants, the graphs also show the results obtained for single adsorbates at the same initial concentration.

**Figure 10:**
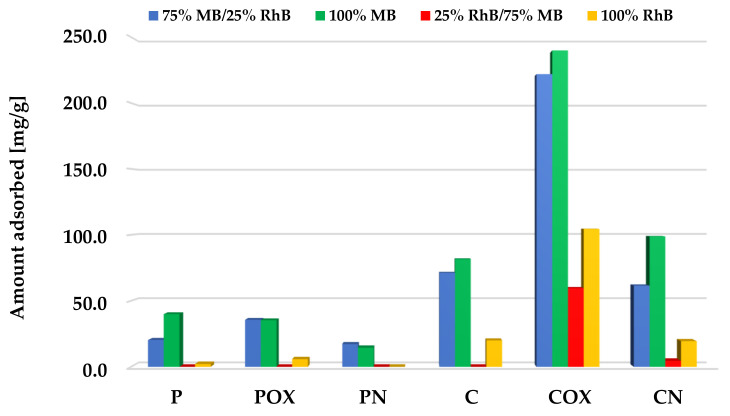
Comparison of the activated biocarbons sorption capacities for the binary and single-adsorbate systems (methylene blue initial concentration significantly higher than for rhodamine B).

**Figure 11:**
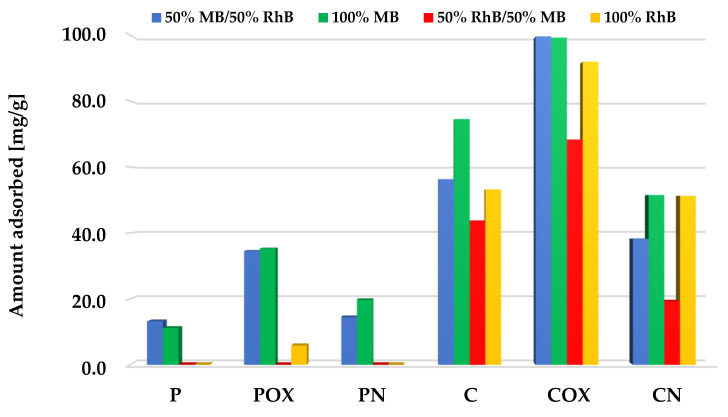
Comparison of the activated biocarbons sorption capacities for the binary and single-adsorbate systems (equal initial concentrations of methylene blue and rhodamine B).

**Figure 12:**
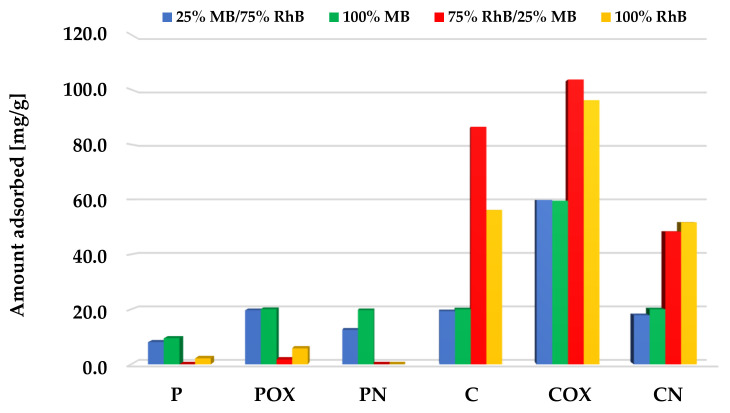
Comparison of the activated biocarbons sorption capacities for the binary and single-adsorbate systems (methylene blue initial concentration significantly lower than for rhodamine B).

According to the data presented in Figure 10, Figure 11 and Figure 12, samples obtained via chemical activation and further modification of elderberry inflorescence showed better sorption abilities towards the mixture of both cationic dyes. The results obtained for the two-component systems clearly indicate that methylene blue and rhodamine B molecules compete with each other for the active sites located on the surface and/or in the pores of the activated biocarbons. This is evidenced by the fact that the sorption capacities towards MB and RhB obtained for systems containing both adsorbates were lower than the analogous results achieved for single adsorbates. 

In case of the systems with a higher initial concentration of methylene blue (Figure 10), adsorption of this dye was preferred. This is evidenced by the fact the obtained sorption capacities were similar or only slightly lower than for the single-component systems. This suggests that the presence of small amounts of rhodamine B did not adversely affect the efficiency of methylene blue removal from the aqueous solutions. A completely opposite tendency of changes was observed in the case of the rhodamine B adsorption. For all the activated biocarbons under investigation, the presence of significant amounts of MB molecules in the system contributed to a drastic decrease in the sorption capacity towards RhB. This was most probably a consequence of the smaller size of the MB molecules, which makes it easier for them to reach the inside part of the porous structure of activated biocarbons.

The situation was quite similar for the systems with equal concentrations of both organic dyes (Figure 11). Also, methylene blue adsorption was the preferred process here, especially in the case of the samples after physical activation, which were characterized by less favorable sorption abilities. However, in case of the samples after chemical activation (in particular, for COX activated biocarbon), adsorption of significant amounts of rhodamine B was also observed. This was most probably a consequence of the fact that the mixture of both cationic dyes contained much less MB molecules than in the previously discussed variant, which is why the porous structure of the samples was not fully saturated by them. 

A completely different situation occurred for the systems with a lower concentration of methylene blue (Figure 12). Samples obtained as a result of physical activation preferred the adsorption of methylene blue over rhodamine B, showing the sorption capacity at a level similar to the systems containing single adsorbates. On the other hand, in case of the C and COX samples, during adsorption from the two-component systems, higher sorption capacities towards rhodamine B were noted than for the systems containing only this dye. Therefore, it can be assumed that the presence of small amounts of MB in the system may have a beneficial effect on the efficiency of RhB adsorption from the aqueous solutions. Perhaps there were mutual interactions between the molecules of both adsorbates, which favored the increased adsorption of the dye with a larger molecule size. The situation was slightly complicated by the behavior of the third of the chemically activated samples—CN, for which a slightly lower sorption capacities toward both dyes were noted. However, further investigation is required to fully elucidate this issue.

According to the data presented in Table 10, the activated biocarbons prepared via the direct and chemical activation of elderberry inflorescence performed quite well in terms of methylene blue and rhodamine B adsorption when compared to the carbonaceous materials obtained from various types of biomass. The most favorable, in this respect, was the COX sample obtained as a result of chemical activation of the precursor with H_3_PO_4_ and enriched in oxygen functional groups by nitric acid oxidation; therefore, further research should focus on optimizing its production procedure. The sorption capacity of this sample toward methylene blue was not as impressive as in the case of the biochar derived from soybean dreg (1274 mg/g) [49], activated carbons obtained from bagasse and cluster stalks (714–847 mg/g) [50] or hydrochar obtained from coffee husks (418 mg/g) [51]; however, it significantly exceeded the results obtained for adsorbents prepared from safflower seed [52], rice straw [53] and commercial activated carbon obtained from peat [54]. The situation was similar in the case of the rhodamine B removal from aqueous solution. For example, the adsorption capacity of the COX sample was higher than for lignocellulose [55] and wood biomass-based activated carbons [56]; however, it was significantly lower than for the activated carbons prepared by activation of rice straw [57], bagasse pith [58] and especially lotus leaves [59].

**Figure 13:**
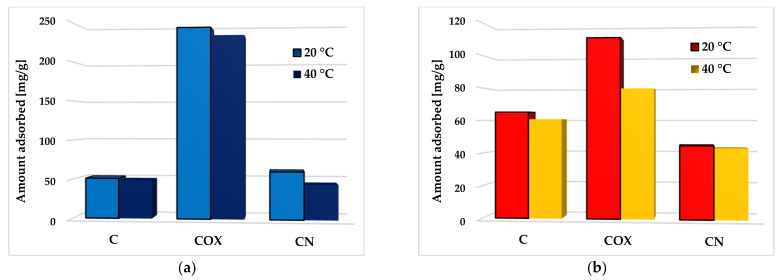
The effect of temperature on methylene blue (**a**) and rhodamine B adsorption (**b**) on the chemically activated biocarbons.

In case of the activated biocarbons obtained as a result of chemical activation and subsequent modifications of elderberry inflorescence, the influence of temperature and pH of the system on the effectiveness of methylene blue and rhodamine B removal from aqueous solutions was also examined. The results concerning the effect of temperature on the process of both organic pollutants adsorption are presented in Figure 13. 

An analysis of the obtained results clearly indicates that temperature of adsorption had a significant effect on the methylene blue and rhodamine B removal efficiency. The adsorption capacity of all activated biocarbon samples decreased with the increasing temperature of the adsorbent–adsorbate system. In the case of MB, the biggest difference in the sorption capacity reached at temperature of 20 °C and 40 °C (by 16.1 mg/g) was observed for the nitrogen-enriched activated biocarbon (CN sample). In turn, in the case of RhB adsorption, the most pronounced changes (by 30.9 mg/g) were observed for the sample COX, enriched in oxygen functional groups. The observed relationship suggests that the interaction between the activated biocarbon surface and methylene blue or rhodamine B molecules was rather exothermic, and their adsorption was mainly based on the physical process, which dominated at lower temperatures.

**Figure 14:**
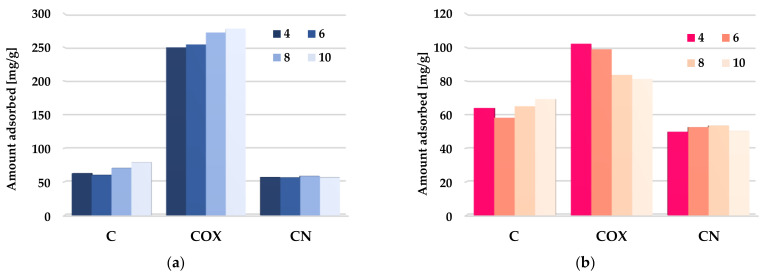
The effect of solution pH on methylene blue (**a**) and rhodamine B adsorption (**b**) on the chemically activated biocarbons.

According to the data presented in Figure 14, the impact of adsorbate pH on the efficiency of methylene blue and rhodamine B removal from aqueous solutions was very diverse. In the case of the unmodified activated biocarbon (C sample), an increase in solution pH from four to six resulted in a slight decrease in the both organic dyes removal efficiency, whereas further change of pH in the range 6–10 led to an improvement of sorption effectiveness. A similar trend of changes was observed in case of the MB adsorption on the COX sample. The increase in pH resulted in an improvement of the adsorption capacity in the entire investigated range, reaching a maximum at pH 10. At low pH values, the majority of the functional groups present on the activated biocarbons surface were protonated, in effect of which the excess of H^+^ ions can compete with the methylene blue and rhodamine B molecules for the access to the active sites. At higher pH values, surface functional groups were deprotonated and negative charge appeared on the adsorbent’s surface. This was conductive to electrostatic interaction between the cationic dyes and activated biocarbon surface. Interestingly, in the case of rhodamine B adsorption on the COX sample, the opposite tendency of changes was observed. This behavior may be related to the fact that RhB can exhibit different molecular forms in different pH solution, i.e., it is in a monomeric form (RhBH^+^) below pH = 3.0 and in a zwitterionic form (RhB^±^) at pH > 4.0. As a result of this, H^+^ ion may compete with cations in the dye solution, while OH^−^ may compete with the anions [60]. A thorough explanation of this issue requires more detailed research. In turn, in the case of nitrogen-enriched activated biocarbon (CN sample), the impact of solution pH on the sorption capacity toward MB and RhB was rather small.

## 3. Materials and Methods

### 3.1. Activated Biocarbons Preparation

**Figure 15:**
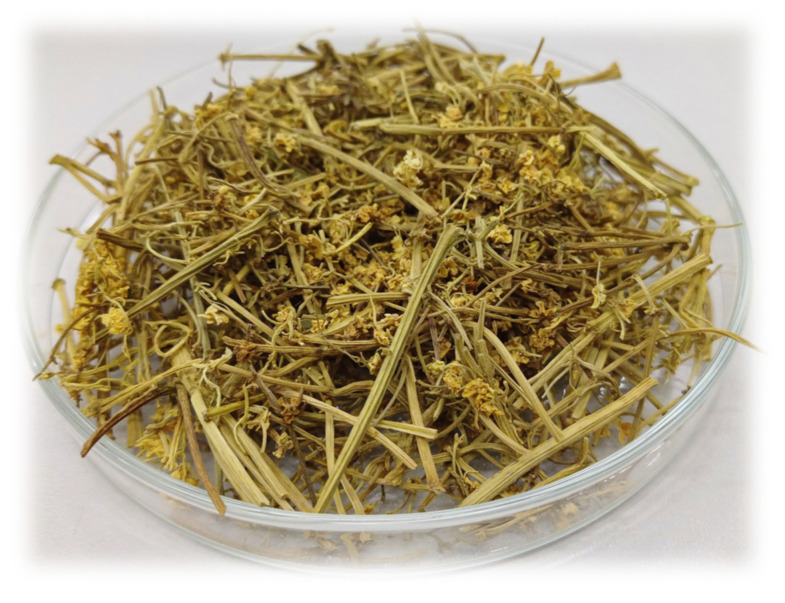
Precursor of the activated biocarbons—elderberry inflorescence.

The elderberry inflorescence (*Sambucus nigra*, Figure 15) used as the starting material for activated biocarbons production came from the Lublin Uplands region (Poland). At the beginning, the precursor was air-dried, crushed and sieved to a size of 5–10 mm. After that, one portion of the fragmented starting material was subjected direct physical activation with CO_2_ (P sample). The remaining part of the precursor was subjected to chemical activation with orthophosphoric acid (C sample). The parameters for the thermochemical treatment of the precursor were selected based on our previous studies, described in detail in [41,61]. Direct activation was carried in the one-heating-zone laboratory furnace, equipped with a quartz tubular reactor (Thermo Fisher Scientific Inc., Waltham, MA, USA). Approximately 15 g of the crushed elderberry inflorescence was placed in the nickel boat and subjected to thermal treatment under carbon dioxide atmosphere (Linde Gaz Polska, Kościan, Poland; flow rate 15 dm^3^/h). The boat with the precursor was placed in the furnace preheated to a temperature of 700 °C and thermostated under these conditions for a period of 30 min. Finally, the sample was taken out from the hot zone of the furnace and cooled down to room temperature.

Chemical activation proceeded according to the following procedure: crushed elderberry inflorescence was impregnated with 50% solution of orthophosphoric acid (STANLAB, Lublin, Poland) at the precursor-activating agent weight ratio equal to 1:2. After 24 h, the sample was dried at 110 °C, placed into the quartz boat and heated in the one-heating-zone horizontal laboratory furnace, provided by Czylok, Jastrzębie-Zdrój, Poland. Thermochemical treatment of the impregnated precursor was carried out under nitrogen atmosphere (technical nitrogen 4.0, Linde Gaz Polska, Kościan, Poland, flow rate 20 dm^3^/h) and consisted of the three stages. Initially, the sample was heated under nitrogen atmosphere to temperature of 200 °C (at the rate of 5 °C/min) and annealed under these conditions for 30 min. In the second stage, the sample was heated to the final activation temperature of 500 °C and again annealed for 30 min. Finally, the sample was cooled down to room temperature, subjected to post-activation washing procedure with hot distilled water and dried at 110 °C. 

In order to obtain materials characterised by diverse textural parameters as well as acidic-basic character of the surface, both prepared activated biocarbons were next subjected to two variants of thermochemical modification: (1) oxidation with boiling 50% solution of nitric acid (Avantor Performance Materials, Gliwice, Poland) for 3 h (denoted as OX) and (2) impregnation with urea (Chempur, Piekary Śląskie, Poland) at the weight ratio of 1:1, followed by heat treatment at 350 °C under nitrogen atmosphere for 2 h (denoted as N).

### 3.2. Physicochemical Characterization of the Precursor and Activated Biocarbons

The elemental analysis of the starting elderberry inflorescence as well as products of its activation and modification was performed using the CHNS Vario EL III instrument (Elementar Analysensysteme GmbH, Langenselbold, Germany). The ash content for all the carbonaceous materials under investigation was determined according to the ISO 1171:2002 Standard, using the microwave muffle furnace (Phoenix model, CEM Corporation, Matthews, IL, USA).

The content of surface functional groups of basic and acidic character was determined according to the Boehm back titration method. Volumetric standards of 0.1 mol/dm^3^ NaOH (POCH S.A., Gliwice, Poland) and 0.1 mol/dm^3^ HCl (POCH S.A., Gliwice, Poland) were used as the titrants. A detailed description of the procedure was presented in our earlier paper [25]. The pH value for the precursor and activated biocarbons aqueous extracts was evaluated using the CP-401 pH-meter (ELMETRON, Zabrze, Poland) equipped with combination glass electrode (accord. to the procedure described in [25]).

Textural characterisation of the elderberry-based activated biocarbons was performed on the Autosorb iQ sorptometer (Quantachrome Instruments, Boynton Beach, FL, USA) at the temperature of –196 °C. Before the nitrogen adsorption—desorption isotherms measurement, the samples were degassed under vacuum at the temperature of 300 °C for 12 h. The specific surface area of activated biocarbons was designated on the basis of the multilayer adsorption BET theory, in the relative pressure (p/p_0_) range of 0.05–0.30. Total pore volume was calculated by converting the amount of nitrogen adsorbed at p/p_0_ = 0.99. Pore size distribution for each carbonaceous material was determined based on the BJH model. *t-plot* method was applied to determine micropore volume and micropore surface area.

Thermal analysis of the activated biocarbons was carried out on a STA 449 Jupiter F1 (Netzsch, Selb, Germany) under the following operational conditions: heating rate 10 °C/min, the dynamic atmosphere of helium (50 cm^3^/min) or air (50 cm^3^/min) in the temperature range of 25–1000 °C, sample mass of about 5 mg, sensor thermocouple type P TG-DSC. As a reference, empty Al_2_O_3_ crucible was used.

### 3.3. Adsorption of Methylene Blue (MB) and Rhodamine B(RhB)

Two synthetic cationic dyes—methylene blue and rhodamine B (Avantor Performance Materials, Gliwice, Poland)—were used in order to characterize the sorption abilities of the elderberry-based activated biocarbons. Adsorption tests were performed according to procedure described in details in our earlier work [26]. The effect of the initial MB and RhB concentration on the removal efficiency of the above-mentioned organic dyes from the water solutions was investigated. Initial and final dyes concentration in the solution were determined using a double beam UV–Vis spectrophotometer Cary 100 Bio provided by Agilent (Santa Clara, CA, USA) at the wavelength of 664 nm (for methylene blue) and 554 nm (for rhodamine B), using the previously prepared calibration curves. Distilled water was applied as a reference sample in both cases.

The amount of adsorbed MB or RhB (q, mg/g) was calculated according to Equation (1) [62]:

(1) $$q=\frac{\Delta c\cdot V_{}}{m}$$

where Δc is the difference between the initial and final dye concentration [mg/dm^3^], V is the volume of MB or RhB aqueous solution used for the adsorption test [dm^3^], m is the mass of activated biocarbon used for the adsorption test [g].

The effectiveness of MB and RhB removal (Ef, %) from the aqueous solutions was calculated according to the following Formula (2) [62]:

(2) $$Ef=\frac{\Delta c}{c_{0}}\cdot 100\%$$

where c_0_ is the starting concentration of methylene blue and rhodamine B [mg/dm^3^].

Two most popular adsorption isotherms models were applied to fit the equilibrium data. These isotherms included the Langmuir and Freundlich model, expressed by Equations (3) and (4) [63,64]:

(3) $$q=\frac{q_{m}K_{L}c}{1+K_{L}c}$$

(4) $$q=K_{F}c^{1/n}$$

where q is the equilibrium MB or RhB adsorbed amount [mg/g], q_m_ is the maximal MB or RhB adsorbed amount [mg/g], K_L_ is the Langmuir constant [dm^3^/mg], c is the equilibrium concentration of MB or RhB [mg/dm^3^], K_F_ is the Freundlich constant [mg/g (mg/dm^3^)^1/*n*^], *n* is the Freundlich parameter determining the adsorption strength.

## 4. Conclusions

The above-discussed results proved that herbal industry waste—such as the elderberry inflorescences—can be successfully used as an alternative and renewable precursors for the production of activated biocarbons. Carbonaceous materials obtained as a result of physical and chemical activation of this type of biomass are characterized by completely diverse physicochemical parameters, which can be additionally modified by means of the further thermochemical treatment; for example, by introducing of oxygen or nitrogen functional groups into carbon matrix. The activated biocarbons obtained in this way differed significantly not only in terms of the elemental composition, thermal stability and chemical nature of the surface (from strongly acidic to alkaline), but also showed a very diverse degree of specific surface development and the type of porous structure generated (from micro/mesoporous to extremely mesoporous). Adsorption tests showed that the obtained carbonaceous materials were also characterized by very diverse sorption abilities in relation to cationic organic dyes. Materials prepared via chemical activation and further modification of the elderberry inflorescence performed much better in terms of removal of organic pollutants from aqueous solutions (both in case of the adsorption from single-component and binary solutions). The most effective adsorbent was the COX sample, obtained by chemical activation of the precursor, followed by oxidation with the nitric acid. Its maximum sorption capacity toward methylene blue and rhodamine B reached the level of 277.8 mg/g and 98.1 mg/g, respectively. The equilibrium analysis reflected that the adsorption of MB and RhB proceeds in accordance with the mechanism proposed by Langmuir, i.e., with the formation of an dye molecules monolayer on the surface of the activated biocarbons. It was also shown that the efficiency of methylene blue and rhodamine B adsorption from aqueous solutions decreased with increasing temperature of the system. In turn, the impact of pH in case of the particular adsorbents and adsorbates was very diverse.

## Figures and Tables

**Table 1:** Elemental composition of the elderberry inflorescence and activated biocarbons [wt.%].

Sample	Ash	C^daf 1^	H^daf^	N^daf^	S^daf^	O^diff 2^
Precursor	9.2	49.1	7.2	2.7	0.6	40.4
P	26.2	75.8	1.2	4.0	0.3	18.7
POX	2.7	69.6	2.4	4.2	0.3	23.5
PN	21.6	83.4	1.6	4.7	0.4	9.9
C	8.5	84.5	3.9	2.0	0.2	9.4
COX	4.3	69.5	1.3	4.1	0.2	24.9
CN	5.9	84.8	3.6	4.6	0.1	6.9

P—physically activated samples; C—chemically activated samples; OX—oxidized biocarbons, N—nitrogen-enriched biocarbons; ^1^ dry-ash-free basis; ^2^ calculated by difference; method error ≤ 0.3%.

**Table 2:** Acidic-basic character of the precursor as well as the activated biocarbons surface.

Sample	Acidic Groups Content [mmol/g]	Basic Groups Content [mmol/g]	Total Content of Surface Groups [mmol/g]	pH of Aqueous Extracts
Precursor	0.74	1.19	1.93	5.09
P	0.00	5.28	5.28	10.99
POX	2.72	0.90	3.62	3.65
PN	0.15	4.17	4.32	10.65
C	1.18	0.60	1.78	2.62
COX	1.27	1.05	2.32	4.67
CN	0.55	1.27	1.82	6.11

**Table 3:** Textural parameters of the activated biocarbons prepared from elderberry inflorescence.

Sample	Total ^1^		Micropore		Micropore Contribution [%]	Mean Pore Size [nm]
	Surface Area [m^2^/g]	Pore Volume [cm^3^/g]	Area [m^2^/g]	Volume [cm^3^/g]		
P	2	0.034	-	-	-	67.280
POX	342	0.275	278	0.155	56.4	3.210
PN	56	0.117	10	0.005	4.3	8.255
C	316	0.460	130	0.070	15.2	5.802
COX	146	0.252	39	0.020	7.9	6.894
CN	333	0.449	167	0.087	19.4	5.389

^1^ method error in the range from 2 to 5%.

**Table 4:** Results of the TG/DTG analysis for samples obtained by physical activation.

Sample	T1/°C	T2/°C	T3/°C	T4/°C	T5/°C	T6/°C	ITD/°C	Residual Mass (%)
P-H	56.6	140.7	551.7	656.2	786.1	931.3	682.8	59.7
POX-H	52.7	276.0	582.6	677.0	--	--	850.0	63.7
PN-H	62.0	--	--	674.2	--	--	746.5	80.2
P-A	67.4	145.1	407.1 465.3	674.7	821.7	--	257.9	29.7
POX-A	71.7	271.6	591.2	--	--	--	364.8	15.8
PN-A	67.7	--	485.0 559.7	658.8	--	--	286.7	26.0

H—in helium atmosphere, A—in air atmosphere.

**Table 5:** Results of the DSC analysis for samples obtained by physical activation.

Sample	D1/°C	D2/°C	D3/°C	D4/°C	D5/°C	ITD/°C
P-H	* 43.6	* 140.9	* 664.1	--	--	772.9
POX-H	* 51.4	* 292.6 * 359.3	* 483.1 * 523.5	* 657.1 * 695.0	--	861.9
PN-H	* 44.0	--	* 476.1 * 536.6	* 675.7	--	865.2
P-A	Below * 80.7	* 149.0	** 417.2, ** 477.6	** 668.6	** 847.9	293.6
POX-A	* 65.7	* 207.4	** 592.8	--	--	282.2
PN-A	Below * 92.8	--	** 479.5, ** 563.6	--	--	290.1

* endothermic effect, ** exothermic effect.

**Table 6:** Results of the TG/DTG analysis for samples obtained by chemical activation.

Sample	T1/°C	T2/°C	T3/°C	T4/°C	T5/°C	ITD/°C	Residual Mass (%)
C-H	47.1	230.4	581.7	657.8	870.0	750.5	79.5
COX-H	57.7	307.9	510.2	583.2	650.8	846.3	57.5
CN-H	53.6	211.2	483.1	577.7	651.0	810.7	81.0
C-A	60.8	223.8	397.5 613.0 713.6	--	--	327.5	9.0
COX-A	73.8	--	295.4 517.9	723.0	--	161.3	9.0
CN-A	47.4	221.3	400.5 622.9	--	--	330.0	5.2

H—in helium atmosphere, A—in air atmosphere.

**Table 7:** Results of the DSC analysis for samples obtained after chemical activation.

Sample	D1/°C	D2/°C	D3/°C	D4/°C	D5/°C	D6/°C	ITD/°C
C-H	* 43.3	* 236.2	--		* 526.3	* 663.3	758.1
COX-H	* 54.5	* 223.0	* 366.7	* 460.8	* 548.6	* 653.5 * 697.8	780.9
CN-H	* 59.6	* 200.4	* 360.3 * 433.3	--	* 567.4 * 609.5 * 665.4	--	770.9
C-A	Below * 102.0	* 283.3	** 602.3 ** 729.6	--	--	--	427.2
COX-A	* 80.8	--	** 513.7 ** 568.1	** 740.5	--	--	232.3
CN-A	* 48.5	--	** 385.2 ** 609.1	--	--	--	285.7

* endothermic effect, ** exothermic effect.

**Table 8:** Langmuir/Freundlich parameters of the isotherms of methylene blue adsorption on the activated biocarbons prepared from elderberry inflorescence.

Sample	q_e_	Langmuir Model			Freundlich Model		
		q_m_	K_L_	R^2^	K_F_	1/n	R^2^
P	11.75	11.59	10.3976	0.9990	8.8064	0.1916	0.6846
POX	35.54	36.90	24.6364	0.9958	32.5013	0.0658	0.8480
PN	14.54	14.66	8.7436	0.9995	10.9597	0.1244	0.8485
C	87.48	86.21	2.1887	0.9977	53.9511	0.2037	0.8307
COX	278.99	277.78	36.0000	0.9883	271.1440	0.2704	0.9235
CN	56.50	55.87	4.9722	0.9982	42.4131	0.1021	0.9558

q_e_—experimental adsorption capacity [mg/g], q_m_—the maximum adsorption capacity [mg/g], K_L_—the Langmuir adsorption equilibrium constant [dm^3^/mg], K_F_—the Freundlich equilibrium constant [mg/g (mg/dm^3^)^1/n^], 1/n—the intensity of adsorption, R^2^—the determination coefficients.

**Table 9:** Langmuir/Freundlich parameters of the isotherms of rhodamine B adsorption on the activated biocarbons prepared from elderberry inflorescence.

Sample	q_e_	Langmuir Model			Freundlich Model		
		q_max_	K_L_	R^2^	K_F_	1/n	R^2^
P	2.79	2.23	2.3996	0.8901	7.2011	0.0890	0.2307
POX	4.75	4.51	0.5985	0.9396	6.6681	0.2215	0.9091
PN	-	-	-	-	-	-	-
C	56.12	56.82	1.5575	0.9982	34.8980	0.1668	0.9726
COX	96.12	99.04	0.0176	0.9944	46.6982	0.3172	0.6727
CN	51.63	52.91	2.9077	0.9977	35.9584	0.1381	0.5773

**Table 10:** Adsorption capacities towards methylene blue and rhodamine B for various adsorbents.

Adsorbent	Maximum Adsorbed Amount [mg/g]	Reference
Methylene blue		
Biochar from soybean dreg	1274	[49]
Activated carbon from bagasse and cluster stalks	714–847	[50]
Hydrochar from coffee husks	418	[51]
Activated carbons from safflower seed	128	[52]
Activated carbon from rice straw	109	[53]
Commercial activated carbon from peat	161	[54]
COX	279	This study
Rhodamine B		
Activated carbon from lignocellulosic waste	33	[55]
Activated carbon from wood biomass	77	[56]
Activated carbon from rice husk	181	[57]
Activated carbon from bagasse pith	199	[58]
Activated carbon from lotus leaves	701	[59]
COX	96	This study

## Data Availability

Data are contained within the article.

## Sample Availability

Samples of biocarbons are available from the authors upon reasonable request.
